# Early exposure of pregnant women to non‐steroidal anti‐inflammatory drugs delivered outside hospitals and preterm birth risk: nationwide cohort study

**DOI:** 10.1111/1471-0528.16670

**Published:** 2021-03-22

**Authors:** C Quantin, C Yamdjieu Ngadeu, J Cottenet, S Escolano, S Bechraoui‐Quantin, P Rozenberg, P Tubert‐Bitter, J‐B Gouyon

**Affiliations:** ^1^ High‐Dimensional Biostatistics for Drug Safety and Genomics Université Paris‐Saclay UVSQ Univ. Paris‐Sud Inserm CESP Villejuif France; ^2^ Biostatistics and Bioinformatics (DIM) University Hospital Dijon France; ^3^ Bourgogne Franche‐Comté University Dijon France; ^4^ Inserm CIC 1432 Dijon France; ^5^ Clinical Investigation Centre Clinical Epidemiology/Clinical Trials Unit Dijon University Hospital Dijon France; ^6^ Department of Obstetrics and Gynaecology Poissy‐Saint Germain Hospital Poissy France; ^7^ Paris Saclay University, UVSQ, Inserm, Team U1018, Clinical Epidemiology, CESP Montigny‐le‐Bretonneux France; ^8^ Centre d’Etudes Périnatales Océan Indien (EA 7388) Centre Hospitalier Universitaire Sud Réunion La Réunion Saint Pierre France

**Keywords:** Early exposure, French National Health Insurance Database, non‐steroidal anti‐inflammatory drugs, risk of prematurity

## Abstract

**Objective:**

To assess the risk of preterm birth associated with nonsteroidal anti‐inflammatory drugs (NSAIDs), focusing on early exposure in the period from conception to 22 weeks of gestation (WG).

**Design:**

National population‐based retrospective cohort study.

**Setting:**

The French National Health Insurance Database that includes hospital discharge data and health claims data.

**Population:**

Singleton pregnancies (2012–2014) with a live birth occurring after 22WG from women between 15 and 45 years old and insured the year before the first day of gestation and during pregnancy were included. We excluded pregnancies for which anti‐inflammatory medications were dispensed after 22WG.

**Methods:**

The association between exposure and risk of preterm birth was evaluated with GEE models, adjusting on a large number of covariables, socio‐demographic variables, maternal comorbidities, prescription drugs and pregnancy complications.

**Main outcome measures:**

Prematurity, defined as a birth that occurred before 37WG.

**Results:**

Among our 1 598 330 singleton pregnancies, early exposure to non‐selective NSAIDs was associated with a significantly increased risk of preterm birth, regardless of the severity of prematurity: adjusted odds ratio (aOR) = 1.76 (95% CI 1.54–2.00) for extreme prematurity (95% CI 22–27WG), 1.28 (95% CI 1.17–1.40) for moderate prematurity (28–31WG) and 1.08 (95% CI 1.05–1.11) for late prematurity (32–36WG), with non‐overlapping confidence intervals. We identified five NSAIDs for which the risk of premature birth was significantly increased: ketoprofen, flurbiprofen, nabumetone, etodolac and indomethacin: for the latter, aOR = 1.92 (95% CI 1.37–2.70) with aOR = 9.33 (95% CI 3.75–23.22) for extreme prematurity.

**Conclusion:**

Overall, non‐selective NSAID use (delivered outside hospitals) during the first 22WG was found to be associated with an increased risk of prematurity. However, the association differs among NSAIDs.

**Tweetable abstract:**

French study for which early exposure to non‐selective NSAIDs was associated with increased risk of prematurity.

## Tweetable abstract

French study for which early exposure to non‐selective NSAIDs was associated with increased risk of prematurity.

## Introduction

Exposure to medication during pregnancy has been shown to be frequent, particularly in France.^1^ Non‐steroidal anti‐inflammatory drugs (NSAIDs) are widely used in the general population and are also prescribed to pregnant women who may need them to treat chronic illnesses or relieve pain (chronic or acute).

The fetal consequences of late exposure to NSAIDs have been documented, particularly for premature closure of the ductus arteriosus. The global fetal toxicity of NSAIDs has led to limitation of their use during pregnancy: non‐selective NSAIDs are generally contraindicated from the 6th month of pregnancy and COX‐2 inhibitors throughout pregnancy (see safety statements for health professionals in France and the USA),^2^, ^3^, ^4^ leading to a significant decrease in NSAID prescription in France in the 3rd trimester.^1^, ^2^, ^3^, ^4^, ^5^

However, the safety of early exposure to NSAIDs in pregnant women has not been fully established, particularly with regard to prematurity. This seems particularly relevant, as prematurity is a frequent and serious condition,^6^ but very few studies have examined early exposure to NSAIDs^7^, ^8^, ^9^, ^10^ and most did not analyse the effect on prematurity as the main study outcome. In early studies where the exposure period was defined as the entire pregnancy, no risk of prematurity was associated with the prescription of NSAIDs (including COX‐2 inhibitors).^7^, ^8^ In contrast, Bérard et al.^9^ recently identified an increased risk of prematurity after exposure to COX‐2 inhibitors in the 3 months prior to delivery, but the authors found no association with exposure during pregnancy up to 3 months prior to delivery.

One physio‐pathological explanation associated to early exposure of pregnancy to NSAIDs could be an utero‐placental hypoperfusion induced by inhibition of prostaglandin synthesis. This pathway has already been suggested as a mechanism of placental mal‐implantation leading to miscarriages associated with early exposure to NSAIDs.^11^, ^12^, ^13^, ^14^

In this study, we assessed the risk of preterm birth associated with NSAIDs delivered outside of hospitals, focusing on a defined period of fixed early exposure from conception to 22 weeks of gestation. The study used the French health insurance database and included nearly 1.6 million pregnant women.

## Methods

### Data sources

The SNDS (Système National des Données de Santé) database was used for this nationwide cohort study. This French national information system contains individual, exhaustive and linked but anonymous data on healthcare use for approximately 97% of the French population. The SNDS aggregates data from two nationwide datasets linked by a unique patient identifier: the French hospital discharge (PMSI – Programme de Médicalisation des Systèmes d’Information) database and the French national health insurance (DCIR – Datamart de Consommation Inter‐Régimes) database.^15^

The PMSI database provides detailed medical information on all admissions to public and private hospitals in France, including discharge diagnosis according to the 10th edition of the International Classification of Diseases (ICD‐10 codes), medical procedures coded according to the French medical classification for clinical procedures (CCAM), and data related to pregnancy, such as gestational age. In particular, almost all deliveries are recorded in the PMSI database because out‐of‐hospital delivery is rare in France, accounting for only 0.4% of all births in 2005–2006.^16^

The DCIR database contains all individualised and anonymous healthcare claims reimbursed outside hospitals by French National Health Insurance. These claims data include dispensed drugs, which are coded according to the Anatomical Therapeutic Chemical (ATC) classification, and outpatient medical procedures, coded according to the CCAM. The DCIR database also collects patient data, such as age, sex and eligibility for complementary universal health insurance (CMU‐C), which provides free access to healthcare for low‐income people.^17^ Eligibility for 100% health insurance coverage for serious and costly long‐term diseases (LTDs), coded according to the ICD‐10, is also recorded in the DCIR database.

Various control procedures are regularly conducted to ensure the quality of these data. For 20 years, hospital data in France have been used for medical research purposes, and the quality of the French hospital database has been confirmed in recent validation and epidemiological studies.^18^, ^19^ Moreover, the SNDS is increasingly used to conduct large‐scale pharmaco‐epidemiological studies, including recent pregnancy studies.^20^, ^21^, ^22^, ^23^, ^24^

### Study design and setting

In this population‐based retrospective cohort study, we included all hospital discharge abstracts for all deliveries (mentioning the ICD‐10 code Z37 ‘outcome of delivery’) that occurred after 22 completed weeks of gestation in France between January 2012 and December 2014. If a woman had several pregnancies during the study period, all of them were taken into account. In France, a first‐trimester ultrasound scan is routinely performed, and used to determine gestational age since the early 2000s. Both the first day of the last menstrual period (first day of gestation: 1DG) and ultrasound are used and information given by last menstrual period may be corrected, in case of discrepancy. The collection of this information in hospital discharge abstracts has been validated by previous studies.^25^, ^26^, ^27^

We included women between 15 and 45 years of age on the 1DG, continuously insured the year before the 1DG and during pregnancy, and who had a live birth occurring after 22WG.

We excluded deliveries with no valid anonymous link established between the mother’s abstract and the child’s abstract non‐singleton pregnancies, as we could not identify easily each newborn, especially for newborns with same gender, and also because of the high risk of premature birth. Finally, pregnancies exposed to NSAIDs after 22WG of pregnancy were excluded from the analysis whatever the exposure during the first 22WG, i.e. even if they were exposed to NSAIDs from conception to 22WG and had an ongoing treatment.

### Outcomes

Prematurity was defined according to the WHO classification as a birth that occurred before 37WG.^28^ We also studied extremely preterm deliveries (before 28WG), moderate preterm deliveries (between 28 and 31WG) and late preterm deliveries (between 32 and 36WG).

### Exposure

Non‐selective NSAIDs and COX‐2 inhibitors were the drugs of interest, defined as having an anti‐inflammatory medication dispensing outside hospital. We focused on early exposure, which is defined as an exposure from conception to 22WG (at least one prescription reimbursed). As we included women who had a live birth occurring after 22WG, all women included were potentially exposed until this term.

Drug exposure was classified according to the ATC classification.^29^ More specifically, we considered the ATC code M01A, which includes all NSAIDs for systemic use. We were also able to identify each NSAID from the 7‐character ATC codes. These sub‐categories correspond to the International Nonproprietary Names (INN).

### Covariates

Although NSAIDs were the drugs of interest, two biological agents, etanercept and infliximab, were also considered as covariates in order to have an exhaustive list of the treatment options for inflammatory diseases. Etanercept and infliximab were identified using ATC codes L04AB01 for and L04AB02, respectively.

Several potential variables associated with prematurity were considered in this study. For each woman, socio‐demographic variables were collected at the 1DG, including maternal age, place of residence (urban or rural) and CMU‐C insurance. In France, CMU‐C is intended for economically disadvantaged populations, and was used in this study as a proxy for family precariousness and socio‐economic vulnerability, which are known to impact the characteristics of pregnancy and its management. We also recovered maternal chronic comorbidities recorded during the 12 months before the 1DG or during pregnancy, in particular hypertension (chronic and pregnancy‐induced) and diabetes mellitus (pre‐existing and gestational), identified through ICD‐10 codes, prescription drugs or LTDs. Autoimmune rheumatic diseases 1 year before or during pregnancy were also identified from LTDs, as NSAIDs are indicated for autoimmune diseases. To take into account factors that may increase the risk of prematurity, we considered, in the year before the 1DG: previous pregnancy, rheumatologist visits, hospitalisations and emergency department (ED) visits; but also current pregnancy complications, including oligohydramnios, placenta abruption and pre‐eclampsia. Finally, we included all prescription drugs, identified from ATC codes, purchased during pregnancy in private pharmacies, whether they were written by hospital or by primary care physicians.

### Statistical analyses

The exposed and non‐exposed groups were compared using Student’s and Chi‐square tests for continuous and categorical variables, respectively.

To study the effect of exposure (non‐selective NSAIDs or COX‐2 inhibitors delivered outside hospitals) on the risk of prematurity, we performed generalised estimating equation (GEE) models adjusting for all covariates identified above. We also conducted sensitivity analyses excluding pre‐eclampsia and placental abruption from the explanatory variables, as they could lie on the causative pathway. The GEE models were used to take into account women who had several pregnancies during the study period (11.68%). Adjusted odds ratios (aORs) with 95% confidence intervals (95% CI) were given.

For exposure, the reference category was pregnancies without exposure to non‐selective NSAID/COX‐2 inhibitors (i.e. non‐exposed group). We also performed a sensitivity analysis depending on the chronic or the episodic use of NSAIDs. We defined chronic use as at least one reimbursed dispensed prescription during the 3 months before the pregnancy and at least one other during at least the first trimester of pregnancy.

We also analysed the association between early exposure to NSAID/COX‐2 inhibitors and the risk of prematurity depending on the severity of prematurity: late preterm (born between 32 and 36 WG), moderate preterm (born between 28 and 31 WG) and extremely preterm (born between 22 and 27WG) were also analysed.

The rate of premature birth was calculated for each of the anti‐inflammatory drugs prescribed in early pregnancy.

As NSAIDs are indicated for autoimmune diseases, we also performed stratified analyses on maternal autoimmune disease status during pregnancy.

Because a history of hypertension or diabetes could increase the risk of prematurity, we also performed an analysis excluding pregnancies with these comorbidities.

All statistical analyses were performed using SAS (SAS Institute Inc., Version 9.4, Cary, NC, USA).

### Ethics

This study was approved by the National Committee for data protection (registration number DE‐2017‐037) and The French Institute of Health Data (INDS, registration number 90, 9 September 2014). Written consent was not required for this study because it was a retrospective study and the national data were anonymous. There was no patient or public involvement.

## Results

We identified a total of 1 675 180 pregnancies with live births that occurred after 22WG in women aged between 15 and 45, in France in 2012–2014. After applying our exclusion criteria, we included 1 505 444 women with 1 598 330 singleton pregnancies (Figure S1). In particular, 13 526 (0.8%) pregnancies were excluded due to an exposure over 22WG to NSAIDs/COX‐2 inhibitors and the two biological agents, etanercept and infliximab.

Among these, 130 815 (8.18%) were exposed to NSAIDs delivered outside hospitals, including 851 COX‐2 inhibitors between 1 and up to 22WG. In the exposed group (NSAIDs/COX‐2 inhibitors), 7492 deliveries were premature (5.73%) compared with 72 825 (4.96%) in the non‐exposed group (*P *< 0.01; Table 1), which represents an increase of 15.5%. A significant difference was observed for each class of prematurity: 0.21 versus 0.16% for extreme prematurity, 0.44 versus 0.38% for moderate prematurity, and 5.07 versus 4.43% for late prematurity (*P* < 0.01 for all).

**Table 1:** Characteristics of pregnancies

	All pregnancies (*n* = 1 598 330)	Non‐exposed to any NSAIDS/COX‐2 (*n* = 1 467 515)	Exposed to NSAIDS/COX‐2 (*n* = 130 815)	*P*‐value
Preterm (between 22 and 36WG), *n* (%)	80 317 (5.03%)	72 825 (4.96%)	7492 (5.73%)	<0.0001
Late preterm (between 32 and 36WG), *n* (%)	71 577 (4.48%)	64 941 (4.43%)	6636 (5.07%)	<0.0001
Moderate preterm (between 28 and 31WG), *n* (%)	6124 (0.38%)	5544 (0.38%)	580 (0.44%)	0.0002
Extremely preterm (between 22 and 27WG), *n* (%)	2616 (0.16%)	2340 (0.16%)	276 (0.21%)	<0.0001
Age at pregnancy (years) – mean ± SD	29.37 ± 5.13	29.37 ± 5.09	29.34 ± 5.50	0.036
CMU‐C insurance, *n* (%)	225 498 (14.11%)	192 498 (13.12%)	33 000 (25.23%)	<0.0001
Rural dweller, *n* (%)	305 452 (19.11%)	283 931 (19.35%)	21 521 (16.45%)	<0.0001
Pregnancy in the year prior to the 1DG of gestation, *n* (%)	185 051 (11.58%)	169 161 (11.53%)	15 890 (12.15%)	<0.0001
Autoimmune rheumatic diseases, *n* (%)	7564 (0.47%)	6760 (0.46%)	804 (0.61%)	<0.0001
Systemic lupus erythematosus, *n* (%)	2295 (0.14%)	2089 (0.14%)	206 (0.16%)	0.1663
Rheumatoid arthritis, *n* (%)	1253 (0.08%)	1081 (0.07%)	172 (0.13%)	<0.0001
Multiple sclerosis, *n* (%)	3225 (0.2%)	2891 (0.2%)	334 (0.26%)	<0.0001
Rare autoimmune diseases, *n* (%)	873 (0.05%)	780 (0.05%)	93 (0.07%)	0.0078
Biological agents * between 1 and 22WG, *n* (%)	341 (0.02%)	292 (0.02%)	49 (0.04%)	<0.0001
Number of other medications during pregnancy, mean ± SD	6.38 ± 3.90	6.19 ± 3.78	8.52 ± 4.53	<0.0001
Prescription for aspirin during pregnancy, *n* (%)	58 710 (3.67%)	53 329 (3.63%)	5381 (4.11%)	<0.0001
Prescription for acetaminophen during pregnancy, *n* (%)	1 070 495 (66.98%)	955 580 (65.12%)	114 915 (87.85%)	<0.0001
Current pregnancy complication, *n* (%)	53 811 (3.37%)	48 872 (3.33%)	4939 (3.78%)	<0.0001
Male infants, *n* (%)	817 527 (51.15%)	750 352 (51.13%)	67 175 (51.35%)	0.1266
Hypertension, *n* (%)	39 539 (2.47%)	34 174 (2.33%)	5365 (4.1%)	<0.0001
Diabetes, *n* (%)	12 591 (0.79%)	11 115 (0.76%)	1476 (1.13%)	<0.0001
Depression, *n* (%)	86 937 (5.44%)	75 489 (5.14%)	11 448 (8.75%)	<0.0001
Asthma, *n* (%)	146 570 (9.17%)	130 082 (8.86%)	16 488 (12.6%)	<0.0001
Thyroid disorders, *n* (%)	49 258 (3.08%)	44 670 (3.04%)	4588 (3.51%)	<0.0001
Rheumatologist visits, *n* (%)	32 256 (2.02%)	27 647 (1.88%)	4609 (3.52%)	<0.0001
Emergency visit and/or hospitalisation, *n* (%)	282 860 (17.7%)	254 859 (17.37%)	28 001 (21.41%)	<0.0001

DG, first day of gestation; SD, standard deviation; WG, weeks of gestation.

Non‐exposed and exposed groups were compared using Student’s *t*‐test (continuous variables) of Chi‐square test (categorical variables).

* Etanercept and infliximab.

Regarding the patient characteristics, women exposed to early exposure to NSAIDs/COX‐2 inhibitors were more likely to be on CMU‐C and living in urban areas. They were more likely to be hospitalised or have ED visits and visits to rheumatologists. They were also more likely to have autoimmune rheumatic diseases such as rheumatoid arthritis, and they had more comorbidities such as hypertension, diabetes, depression or anxiety, and asthma. They were also more likely to have had a pregnancy in the previous year (Table 1).

After adjustment for risk factors and potential confounders (Table 2), non‐selective NSAIDs were associated with an increased risk of prematurity, either alone (aOR = 1.08, 95% CI 1.06–1.11) or combined with a biological agent, etanercept or infliximab (aOR = 3.16, 95% CI 1.37–7.28). After dropping pre‐eclampsia and placental abruption, non‐selective NSAIDs remained associated with prematurity (aOR = 1.08, 95% CI 1.06–1.11). In the sensitivity analysis for the chronic and the episodic use of NSAIDs, we found that the risk of prematurity was the same whatever the use of non‐selective NSAIDs: aOR = 1.08 (95% CI 1.04–1.13) for chronic use and aOR = 1.08 (95% CI 1.04–1.11] for episodic use.

**Table 2:** GEE Models: adjusted odds ratios with 95% confidence intervals for the risk of premature birth after exposure (non‐selective NSAIDs, COX‐2 inhibitors)

	All (22 to <37WG, *n* = 80 317)	Extremely preterm (22 to <28WG, *n* = 2616)	Moderate preterm (28 to <32WG, *n* = 6124)	Late preterm (32 to < 37WG, *n* = 71 577)
Exposure to study medications between 1 and 22WG				
Non‐selective NSAIDs only	1.08 (1.05–1.11)	1.76 (1.54–2.00)	1.28 (1.17–1.40)	1.08 (1.05–1.11)
COX‐2 inhibitors only	0.79 (0.57–1.10)	–	–	0.91 (0.65–1.27)
Combined COX‐2 inhibitors and non‐selective NSAIDs/biological agents*	1.26 (0.81–1.96)	4.58 (0.64–32.89)	2.35 (0.62–8.96)	1.19 (0.74–1.93)
Combined non‐selective NSAIDs and biological agents	3.16 (1.37–7.28)	–	4.80 (0.49–47.07)	2.75 (1.15–6.58)
Treatment options for inflammatory diseases: biological agents only	1.52 (0.98–2.36)	1.68 (0.23–12.59)	1.01 (0.23–4.53)	1.50 (0.96–2.34)
Demographic characteristics at 1DG				
Age at pregnancy	1.00 (0.999–1.002)	0.99 (0.98–1.000)	1.00 (0.994–1.004)	1.00 (0.997–1.000)
CMU‐C insurance	1.38 (1.36–1.41)	1.89 (1.71–2.08)	1.58 (1.48–1.68)	1.34 (1.31–1.37)
Rural dweller	0.98 (0.97–1.003)	0.73 (0.65–0.82)	0.88 (0.82–0.94)	1.00 (0.98–1.02)
Pregnancy in the year prior to the 1DG of gestation	1.05 (1.02–1.07)	1.11 (0.98–1.27)	1.06 (0.97–1.15)	1.02 (0.99–1.05)
At least 1 diagnostic prior to and/or during pregnancy				
Systemic lupus erythematosus	3.30 (2.93–3.71)	2.80 (1.28–6.12)	2.72 (1.72–4.31)	1.70 (1.44–2.01)
Rheumatoid arthritis	1.36 (1.09–1.70)	–	1.48 (0.76–2.89)	1.23 (0.97–1.55)
Multiple sclerosis	1.38 (1.20–1.59)	1.23 (0.55–2.75)	1.30 (0.78–2.19)	1.38 (1.19–1.60)
Rare autoimmune diseases	1.98 (1.60–2.44)	1.87 (0.79–4.45)	3.14 (2.02–4.88)	1.35 (1.06–1.71)
Number of other medications during pregnancy	0.99 (0.985–0.989)	0.83 (0.82–0.85)	0.92 (0.91–0.92)	0.99 (0.991–0.996)
Current pregnancy complication	7.11 (6.96–7.27)	11.97 (10.97–13.06)	17.38 (16.47–18.34)	5.41 (5.28–5.54)
Male infants	1.17 (1.15–1.19)	1.15 (1.06–1.24)	1.19 (1.13–1.25)	1.16 (1.15–1.18)
Comorbidities or health care utilisation in the year prior to the 1DG				
Hypertension	1.58 (1.53–1.64)	1.53 (1.28–1.82)	1.63 (1.46–1.82)	1.44 (1.39–1.50)
Diabetes	2.59 (2.46–2.74)	2.49 (1.93–3.21)	1.64 (1.36–1.97)	2.53 (2.39–2.67)
Depression	1.22 (1.19–1.26)	1.20 (1.04–1.43)	1.21 (1.09–1.34)	1.22 (1.18–1.26)
Asthma	1.08 (1.06–1.11)	1.17 (1.02–1.33)	1.21 (1.12–1.32)	1.07 (1.04–1.09)
Thyroid disorders	1.02 (0.98–1.06)	0.97 (0.77–1.22)	1.03 (0.89–1.18)	1.01 (0.96–1.05)
Rheumatologist visits	1.06 (1.01–1.12)	1.11 (0.86–1.44)	1.13 (0.96–1.34)	1.04 (0.99–1.10)
Emergency visit and/or hospitalisation	1.20 (1.17–1.22)	1.29 (1.16–1.45)	1.25 (1.16–1.34)	1.18 (1.15–1.20)

aOR, adjusted odd ratio; CI, confidence interval; DG: first day of gestation; GEE, generalised estimating equation; WG: weeks of gestation.

* Biological agents : etanercept and infliximab.

The risk of prematurity was also increased for many factors: CMU‐C, pregnancy in the previous year, hospitalisation or ED visits in the year prior to the pregnancy, comorbidities in the year prior to the pregnancy such as diabetes (aOR = 2.59, 95% CI 2.46–2.74], lupus (aOR = 3.30, 95% CI 2.93–3.71), other autoimmune rheumatic disease, and current pregnancy complications (aOR = 7.11, 95% CI 6.96–7.27).

For women exposed to non‐selective NSAIDs alone, the risk was highest for extreme prematurity (aOR = 1.76, 95% CI 1.54–2.00) and decreased with the degree of prematurity, but it remained significant with an aOR of 1.28 (95% CI 1.17–1.40) for moderate prematurity and 1.08 (95% CI 1.05–1.11) for late prematurity.

After adjustment for the same covariates (Table 3), the combination of at least one non‐selective NSAID and a COX‐2 inhibitor during early exposure increased the risk of prematurity (aOR = 1.13, 95% CI 1.04–1.22). Differentiating between the different NSAIDs, we showed that ketoprofene, flurbiprofene, nabumetone, etodolac and indomethacin were each associated with an increased risk of prematurity after early exposure (Table 3). Women who were prescribed indomethacin more frequently had severe conditions when compared with flurbiprofen, but we found no major differences regarding the conditions when comparing indometacine with diclofenac or other NSAIDs. Moreover, the association between indomethacin and prematurity was still significant after adjusting for these conditions.

**Table 3:** GEE Models: association between the use of different NSAIDs between 1 and 22WG and risk of having a premature birth (aOR with 95% CI)

	*N*	All (22 to <37WG, *n* = 80 317)	Extremely preterm (22 to <28WG, *n* = 2616)	Moderate preterm (28 to <32WG, *n* = 6124)	Late preterm (32 to <37WG, *n* = 71 577)
Prescription of two different NSAIDs	10 288	1.13 (1.04–1.22)	1.61 (1.05–2.45)	1.22 (0.91–1.62)	1.10 (1.01–1.20)
Prescription of a biological agents* and a non‐selective NSAIDs or a COX‐2 inhibitor	49	2.44 (1.05–5.67)	–	3.16 (0.32–30.94)	2.32 (0.99–5.54)
Aceclofenac (M01AB16)	1145	1.21 (0.95–1.55)	2.14 (0.80–5.72)	1.08 (0.44–2.64)	1.18 (0.91–1.52)
Alminoprofene (M01AE16)	31	1.15 (0.26–5.03)	–	–	1.29 (0.30–5.57)
Celecoxib (M01AH01)	656	0.76 (0.51–1.11)	–	–	0.86 (0.59–1.26)
Diclofenac (M01AB05 or M01AB55)	9800	1.03 (0.95–1.13)	1.38 (0.90–2.12)	1.23 (0.92–1.64)	1.00 (0.91–1.10)
Etodolac (M01AB08)	423	1.66 (1.17–2.35)	1.40 (0.19–10.40)	1.10 (0.29–4.23)	1.68 (1.17–2.41)
Etoricoxib (M01AH05)	203	0.93 (0.48–1.79)	–	–	1.02 (0.53–1.97)
Fenoprofene (M01AE04)	101	1.10 (0.46–2.63)	7.41 (0.98–55.98)	–	1.01 (0.40–2.56)
Flurbiprofene (M01AE09)	9259	1.25 (1.15–1.37)	1.41 (0.91–2.17)	1.62 (1.26–2.09)	1.20 (1.10–1.31)
Ibuprofene (M01AE01)	54 420	1.04 (0.99–1.08)	1.18 (0.96–1.44)	1.08 (0.94–1.23)	1.02 (0.98–1.07)
Indomethacin (M01AB01)	377	1.92 (1.37–2.70)	9.33 (3.75–23.22)	4.71 (2.16–10.24)	1.38 (0.92–2.09)
Ketoprofene (M01AE03)	17 583	1.09 (1.02–1.17)	1.50 (1.09–2.07)	1.12 (0.90–1.40)	1.07 (1.003–1.15)
Mefenamique acid (M01AG01)	1170	1.17 (0.91–1.49)	0.51 (0.07–3.58)	0.60 (0.19–1.88)	1.23 (0.96–1.58)
Morniflumate (M01AX22)	840	0.71 (0.49–1.03)	–	0.69 (0.17–2.87)	0.73 (0.50–1.07)
Nabumetone (M01AX01)	947	1.29 (1.001–1.66)	0.70 (0.10–5.00)	0.74 (0.23–2.38)	1.35 (1.05–1.75)
Naproxene (M01AE02)	5473	1.09 (0.97–1.22)	1.44 (0.81–2.56)	1.02 (0.67–1.57)	1.08 (0.95–1.22)
Niflumique acid (M01AX02)	2927	1.12 (0.96–1.31)	1.11 (0.46–2.67)	0.93 (0.51–1.72)	1.13 (0.96–1.34)
Oxicams only (piroxicam /meloxicam/tenoxicam)	3567	1.08 (0.93–1.25)	1.70 (0.88–3.26)	0.78 (0.43–1.43)	1.78 (0.93–1.26)
Phenylbutazone (M01AA01)	7	3.33 (0.41–27.36)	–	–	3.62 (0.43–30.61)
Tiaprofenique acide (M01AE11)	10 085	1.05 (0.96–1.15)	1.68 (1.13–2.49)	0.76 (0.53–1.10)	1.05 (0.96–1.15)

aOR, adjusted odd ratio; CI, confidence interval; GEE, generalised estimating equation; WG, weeks of gestation.

Adjusted for demographic characteristics (age at pregnancy, CMU‐C insurance, rural dweller, pregnancy in the year prior to the 1DG of gestation), diagnostic prior to and/or during pregnancy (systemic lupus erythematosus, rheumatoid arthritis, multiple sclerosis, rare autoimmune diseases, number of other medications during pregnancy, current pregnancy complication, male infants), comorbidities in the year prior to the 1DG (hypertension, diabetes, depression, asthma, thyroid disorders) and healthcare utilisation in the year prior to the 1DG (rheumatologist visits, emergency visit and/or hospitalisation).

* Biological agents : etanercept and infliximab.

When analyses were restricted to pregnant women without autoimmune diseases (Table 4), the results were similar to the main analysis, indicating that early exposure to non‐selective NSAIDs was associated with a 1.08‐fold increased risk of premature birth (aOR = 1.08, 95% CI 1.05–1.11). In the analysis restricted to pregnant women with autoimmune diseases (Table 4), the exposure to non‐selective NSAIDs in early pregnancy was not associated with the risk of premature birth (aOR = 1.04, 95% CI 0.81–1.33).

**Table 4:** GEE Models: stratified analyses on maternal autoimmune disease status during pregnancy. Adjusted odds ratios with 95% confidence intervals for the risk of premature birth (22 to <37WG) after exposure (non‐selective NSAIDs, COX‐2 inhibitors)

	Without autoimmune disease (*n* = 1 590 681)	With autoimmune disease (*n* = 7649)
Exposure to study medications between 1 and 22WG		
Non‐selective NSAIDs only	1.08 (1.05–1.11)	1.04 (0.81–1.33)
COX‐2 inhibitors only	0.79 (0.57–1.11)	0.44 (0.05–3.90)
Combined COX‐2 inhibitors and non‐selective NSAIDs/biological agents*	1.26 (0.81–1.96)	–
Combined non‐selective NSAIDs and biological agents	3.21 (1.39–7.38)	2.07 (0.56–7.69)
Treatment options for inflammatory diseases		
Biological agents only	1.52 (0.98–2.36)	0.44 (0.10–1.93)
Demographic characteristics at 1DG		
Age at pregnancy	1.00 (0.999–1.002)	0.99 (0.98–1.01)
CMU‐C insurance	1.38 (1.36–1.41)	1.12 (0.87–1.43)
Rural dweller	0.98 (0.97–1.003)	0.88 (0.72–1.07)
Pregnancy in the year prior to the 1DG of gestation	1.05 (1.02–1.07)	1.22 (0.95–1.56)
Number of other medications during pregnancy	0.987 (0.985–0.989)	1.02 (0.998–1.033)
Current pregnancy complication	7.11 (6.96–7.27)	7.64 (6.17–9.45)
Male infants	1.17 (1.15–1.19)	1.02 (0.88–1.19)
Comorbidities in the year prior to the 1DG		
Hypertension	1.58 (1.53–1.64)	2.32 (1.81–2.97)
Diabetes	2.59 (2.46–2.74)	1.92 (1.15–3.21)
Depression	1.22 (1.19–1.26)	1.06 (0.83–1.36)
Asthma	1.08 (1.06–1.11)	1.00 (0.78–1.28)
Thyroid disorders	1.02 (0.98–1.06)	0.99 (0.71–1.37)
Healthcare utilisation in the year prior to the 1DG		
Rheumatologist visits	1.06 (1.01–1.12)	0.99 (0.80–1.23)
Emergency visit and/or hospitalisation	1.20 (1.17–1.22)	1.37 (1.15–1.64)

aOR, adjusted odd ratio; CI, confidence interval; DG, first day of gestation; GEE, generalised estimating equation; WG, weeks of gestation.

* Biological agents: etanercept and infliximab.

Finally, excluding pregnancies with prior history of hypertension or diabetes, we found that non‐selective NSAIDs were still associated with an increase in prematurity (aOR = 1.09, 95% CI 1.06–1.12).

## Discussion

### Main findings

In this nationwide cohort study, the frequency of prematurity was 15.5% higher following maternal exposure to at least one NSAID (including COX‐2 inhibitors) delivered outside hospitals during the first 22WG, as compared with the non‐exposed population. Exposure to non‐selective NSAIDs in the first 22WG was associated with a significant increase in preterm births after adjustment for socio‐demographic characteristics and prior conditions (aOR = 1.08, 95% CI 1.05–1.11). This result is the same for both chronic and episodic treatment. Moreover, non‐selective NSAID exposure had a more significant impact on extreme prematurity (22–27WG) than on moderate (28–31WG) or late prematurity (32–36WG), with corresponding aOR of 1.76 (95% CI 1.54–2.00), 1.28 (95% CI 1.17–1.40) and 1.08 (95% CI 1.05–1.11), respectively, and with non‐overlapping confidence intervals. This is an important finding, as survival rates in France are 50% at 22–26WG, 80–85% at 27–28WG, 90–95% at 29–30WG, 95% at 31WG, and 98–99% at 32–34WG.^30^

Drug‐specific analyses were made possible by the large size of the population. A significant association between the exposure to NSAIDs and preterm delivery was limited to five of the 19 NSAIDs prescribed in the first 22WG, i.e. ketoprofen, flurbiprofen, nabumetone, etodolac, and indomethacin when prescribed alone (aOR = 1.92, 95% CI 1.37–2.70, for the latter). Early exposure to selective COX‐2 inhibitors (celecoxib or etoricoxib) did not increase the risk of preterm birth, nor did the two biological agents prescribed alone, etanercept and infliximab, in contrast to the non‐selective NSAIDs. It is worth noting that combination of a non‐selective NSAID and a biological agent was rare (42 pregnancies), but the associated risk of preterm delivery was highest in this cohort (aOR = 3.16, 1.37–7.28).

### Strengths and limitations

The French nationwide database used here has strengths in terms of size (1.6 million pregnancies included), nature and data quality.^25^, ^31^, ^32^, ^33^ The main outcome, prematurity, was based on gestational age, which is a variable that has been previously validated.^25^, ^26^, ^27^ As we excluded pregnancies during which anti‐inflammatory medications were dispensed after 22WG, the exposure period was the same for premature and not premature deliveries.

Because our objective was to study the impact of NSAIDs on prematurity, the study population was restricted to women at risk of prematurity, and we excluded pregnancies that ended in miscarriage or fetal death. This may have resulted in a slight bias in the estimation of the risk of prematurity, which should not alter the conclusion of the study.

We could not take into account all variables associated with prematurity such as race/ethnicity, education level, smoking during pregnancy, number of previous miscarriages, average daily caffeine intake during pregnancy, number of previous pregnancies, or history of fertility problems. As a consequence, unmeasured confounders may exist, which limits the interpretation in terms of causal relationships. In particular, we could not control directly for the indication of use of NSAIDs, such as inflammatory disorders, fever or pain. Nevertheless, major maternal conditions were accounted for in the analysis, including maternal chronic conditions and prescribed medications. Stratified analyses on maternal autoimmune diseases, provided similar results and confirmed the increased risk of prematurity in exposed women without autoimmune diseases. This increased risk was also observed in exposed women without hypertension or diabetes. We were also able to identify chronic versus episodic use of NSAIDS, and we found the same risk of prematurity for both types of use. Although based on a broad definition of chronic treatment, these results are fairly consistent with the fact that adjustment reasonably controlled for confusion by indication. However, we acknowledge the potential for residual confounding by indication.

Another limitation is that we do not have data on the drugs given at hospitals. Furthermore, the SNDS database does not provide information for identifying spontaneous versus induced preterm births, but we can assume that in clinical practice, in France, extreme prematurity is very rarely induced. It is, therefore, likely that this limitation had very little impact on this major effect.

Concerning the bias associated with over‐the‐counter (OTC) administration, NSAIDs can only be obtained in France on prescription for 15 of the 19 INNs prescribed in this study. For example, ibuprofen is widely used in France over the counter. However, among the five NSAIDs for which the risk of premature birth was significantly increased, only one NSAID can be purchased OTC (ketoprofen). Two earlier studies are quite reassuring in that they identified a high compliance rate of 90% in pregnant women^34^ and a self‐treatment rate of only 7.5%.^35^ In addition, the risk of OTC use and subsequent misclassification into the unexposed group would only dilute the findings.

### Interpretation

The overall odds ratio of 1.08 may seem weak, but our study shows that the risk is much higher for extreme prematurity (1.76) and for five specific NSAIDs. This global figure is coherent with a similar increase in preterm birth risk of 1.09 (95% CI 1.02–1.17) in NSAID‐treated pregnant Norwegian women.^10^ Similar results were obtained in Denmark when the rate of preterm deliveries among 17 259 pregnancies not exposed to NSAIDs was compared with 1462 women who were given non‐selective NSAIDs at any time from 30 days before conception to term.^7^

Bérard et al. observed that late administration of celecoxib was responsible for an increase in prematurity in Canada^9^ but did not identify the effect of an earlier exposure to NSAIDs defined as exposure from the first day of gestation up to 3 months before delivery. The prescription conditions were not similar in the Canadian study and in the present study, as the study periods were different (1998–2009 versus 2012–2014), and our study was carried out at a time when there was a strong medical communication about the dangers of using NSAIDs during the third trimester of pregnancy.

The physiopathological considerations are confusing since NSAIDs are well‐known tocolytic agents which direct fetal side effects in late pregnancy justify the global contraindication of NSAIDs in this period (FDA). However, some observations suggest that NSAIDs have a direct action effect on placenta circulation. Placental insufficiency, which is similar to fetal growth restriction, could have a central role in preterm delivery whatever its cause through vascular alterations (utero‐placental ischaemia).^36^, ^37^ A recent study of placental histology in preterm newborns with GA <34 weeks and BW <2000 g^38^ showed that 75% of placentas had at least one feature of maternal vascular malperfusion, illustrating that placental vascular malperfusion is very frequently associated with different categories of preterm birth. However, its precise role as a major risk factor of prematurity is not fully demonstrated, nor is the putative worsening role of NSAIDs. Prospective studies focused on the placenta (histology, receptors, cytokines) should give more insight into the potential impact of NSAIDs. Finally, it is worth noting that the large sample size in this study may make even small differences significant, and therefore this conclusion should be investigated with further studies before it is translated into clinical practice.

## Conclusions

Non‐selective NSAID use (delivered outside hospitals) during the first 22 weeks of pregnancy was found to be associated with an increased risk of prematurity, and particularly extreme prematurity. However, the risk of prematurity differed according to the NSAID. This study suggests that there is an urgent need for complementary investigations, as prematurity is a frequent event, with high rates of mortality and morbidity at the lowest gestational ages, and that an assessment by INN should be preferred to assessment for the whole ATC group. These results should not be overestimated because of the risk of unjustified maternal anxiety and discontinuation of useful treatments, but neither should they be underestimated. Of course, the risk–benefit ratio between the value of these useful treatments in clinical practice and the risk of prematurity needs to be confirmed. Additional comprehensive studies about the physiopathological consequences of prostaglandin synthesis inhibition by non‐selected NSAIDs and Cox‐2 inhibitors are necessary to refine the period during which these treatments may be contraindicated. The results of this study should, therefore, be considered as a pharmaco‐epidemiological warning.

### Disclosure of interests

The Authors report no conflict of interests.

### Contribution to authorship

CQ, SE, PTB, JBG conceived and designed the study. CQ, PTB, JBG supervised the study. JBG was responsible for literature review. CQ, CYN, JC acquired and analysed the data. CYN did the statistical analysis. CQ, CYN, SE, JC, SBQ, PR, PTB, JBG interpreted the data, were involved in preparing the manuscript and contributed to the critical revision of the manuscript. PTB and JBG contributed equally to this paper. The corresponding author attests that all listed authors meet authorship criteria. All authors accept responsibility for the paper as published.

### Details of ethics approval

This study was approved by the National Committee for data protection (registration number DE‐2017‐037) and The French Institute of Health Data (INDS, registration number 90, 9 September 2014). Written consent was not required for this study because it was a retrospective study and the national data were anonymous. As a consequence, the research involves no risk to the subjects; the waiver or alteration will not adversely affect the rights and welfare of the subjects; the research could not practicably be carried out without the waiver or alteration (i.e. it is impracticable to get the consent of the subjects).

This work was supported by the National Research Agency (Grant number: ANR‐15‐CE36‐0006‐01) and the Medical Research Foundation. These French agencies had no role in the collection, analysis, interpretation of data or the writing of the manuscript. The researchers confirm their independence from funders.

### Funding

This work was supported by the National Research Agency (Grant number: ANR‐15‐CE36‐0006‐01) and the Medical Research Foundation.

## Supporting information

**Figure S1**. Cohort selection.

Supplementary Material

Supplementary Material

Supplementary Material

Supplementary Material

Supplementary Material

Supplementary Material

Supplementary Material

Supplementary Material

## Data Availability

The database was transmitted by the National Health Insurance Fund‐CNAM (Caisse nationale de l’assurance maladie). The use of these data by our department was approved by the National Committee for data protection. We are not allowed to transmit these data. Data used in this study are available for researchers who meet the criteria for access to these French data from the National Health Insurance Fund (training that opens a personal accreditation, approval of the protocol by required authorities (Expert Committee to research, studies and evaluations in the health field‐CEREES, and National Committee for data protection‐CNIL) according to the ‘Décret n° 2016‐1872 du 26 décembre 2016 modifiant le décret n° 2005‐1309 du 20 octobre 2005 pris pour l’application de la loi n° 78‐17 du 6 janvier 1978 relative à l’informatique, aux fichiers et aux libertés’ (https://www.legifrance.gouv.fr/eli/decret/2016/12/26/2016‐1872/jo/texte).
