# Supplementary material for: Early exposure of pregnant women to non‐steroidal anti‐inflammatory drugs delivered outside hospitals and preterm birth risk: nationwide cohort study
Source: BJOG. 2021 Mar 22;128(10):1575–84. doi: 10.1111/1471-0528.16670 (PMC8451913; doi:10.1111/1471-0528.16670)
Supplement: Supplementary file 1 — Figure S1. Cohort selection. [file BJO-128-1575-s006.docx]

**Figure S1: Cohort selection**

**1 675 180 pregnancies**

*ending between January 2012 and December 2014 with livebirth after 22 weeks of gestation.*

*23 696 (1.41%) pregnancies with no linkable baby for the index stay*

**1 651 484 pregnancies**

*with at least one linkable baby*

*39 628 (2.40%) multiple pregnancies*

**1 611 856 singleton pregnancies**

*13 238 pregnancies with NSAIDs/COX-2 inhibitors or 294 with biological agents late exposure excluded: overall, 13 526 (0.84%) pregnancies were excluded (among which110 pregnancies with COX-2 inhibitors)*

**1 598 330 singleton pregnancies**

*included in the study cohort*

**1 467 515 (91.82%) pregnancies**

*Non-exposed* to non-selective NSAIDs/COX-2 inhibitors between 1-22 weeks

**130 815 (8.18%) pregnancies**

exposed to non-selective NSAIDs/COX-2 inhibitors between 1-22 weeks:

129,609 to non-selective NSAIDs only 861 to COX-2 inhibitors only

42 to combined non-selective NSAIDs and etanercept or infliximab

303 to combined COX-2 inhibitors and non-selective NSAIDs/etanercept/infliximab

**72 825 (4.96%) pregnancies**

*ending before the completed 37 weeks of gestation,*

**64 941 (4.43%) pregnancies**

*ending between 32 to <37 weeks of gestation,*

**5544 (0.38%) pregnancies**

*ending between 28 to <32 weeks of gestation,*

**2340 (0.16%) pregnancies**

*ending before 28 completed weeks of gestation*

**7492 (5.73%) pregnancies**

*ending before the completed 37 weeks of gestation,*

**6636 (5.07%) pregnancies**

*ending between 32 to <37 weeks of gestation,*

**580 (0.44%) pregnancies**

*ending between 28 to <32 weeks of gestation,*

**276 (0.21%) pregnancies**

*ending before 28 completed weeks of gestation*
